# Fluid management in major burn injuries

**DOI:** 10.4103/0970-0358.70715

**Published:** 2010-09

**Authors:** Mehmet Haberal, A. Ebru Sakallioglu Abali, Hamdi Karakayali

**Affiliations:** Department of General Surgery and Burn and Fire Disasters Institute, Ankara, Turkey

**Keywords:** Severe burns, burn shock, fluid resuscitation

## Abstract

It is a widely accepted fact that severe fluid loss is the greatest problem faced following major burn injuries. Therefore, effective fluid resuscitation is one of the cornerstones of modern burn treatment. The aim of this article is to review the current approaches available for modern trends in fluid management for major burn patients. As these current approaches are based on various experiences all over the world, the knowledge is essential to improve the status of this patient group.

## INTRODUCTION

Appropriate fluid management of major burns directly improves the survival rates of burn patients. Despite the vast array of experience, there are still controversies regarding the best type of fluid management in major burns in the first 24 hours after injury. Currently, fluid resuscitation formulas which were developed over 30 years ago, have been accepted as guidelines, but ongoing studies are focussed on the growing concerns that burn patients are being over-or under fluid-resuscitated, often with indistinct and inappropriate end-point targets.[1] The aim of this article is to review the current approaches available for modern trends in fluid management for major burn patients.

### Pathophysiology of burn shock

Major burn injuries result in an area of necrotic zone, beneath this lies the zone of stasis and results in release of inflammatory mediators (e.g. histamine, prostaglandins, thromboxane, nitric oxide) that increase capillary permeability and lead to localised burn wound oedema.[2,3] This occurs within minutes to hours after injury and is followed by the production of highly reactive oxygen species (ROS) during reperfusion of ischaemic tissues.[4–6] ROS are toxic cell metabolites that include oxygen free radicals and cause local cellular membrane dysfunction and propagate an immune response. Subsequently, the decrease in cellular transmembrane potential is observed in both injured and uninjured tissue. Cellular membrane dysfunction leads to the distribution of sodium-ATPase activity. As such, burn shock, which is a combination of distributive, hypovolemic and cardiogenic shock, begins at the cellular level. Disruption of sodium-ATPase activity presumably causes an intracellular sodium shift which contributes to hypovolemia and cellular oedema.[2,3] Heat injury also initiates the release of inflammatory and vasoactive mediators. These mediators are responsible for local vasoconstriction, systemic vasodilation, and increased transcapillary permeability. Increase in transcapillary permeability results in a rapid transfer of water, inorganic solutes, and plasma proteins between the intravascular and interstitial spaces. Subsequently, intravascular hypovolemia and haemoconcentration develop and maximum levels are reached within 12 hours after injury. The steady intravascular fluid loss due to these sequences of events requires sustained replacement of intravascular volume in order to prevent end-organ hypoperfusion and ischaemia.[7,8] Reduced cardiac output is a hallmark in this early post-injury phase. The reduction in cardiac output is the combined result of decreased plasma volume, increased afterload and decreased cardiac contractility, induced by circulating mediators.[9]

As mentioned above, during this early period in which various pathopysiological changes take place, appropriate fluid management plays a fundamental role.

## FLUID MANAGEMENT

The goal of fluid management in major burn injuries is to maintain the tissue perfusion in the early phase of burn shock, in which hypovolemia finally occurs due to steady fluid extravasation from the intravascular compartment.

### Current approaches to fluid management: Optimal route and necessity of formal resuscitation

Burn injuries of less than 20% are associated with minimal fluid shifts and can generally be resuscitated with oral hydration, except in cases of facial, hand and genital burns, as well as burns in children and the elderly. As the total body surface area (TBSA) involved in the burn approaches 15–20%, the systemic inflammatory response syndrome is initiated and massive fluid shifts, which result in burn oedema and burn shock, can be expected. The route for fluid management is of importance in these instances. Although enteral resuscitation has been attempted for even major burn injuries, vomiting has been a limiting problem for this route.[10] Current recommendations are to initiate formal intravascular fluid resuscitation when the surface area burned is greater than 20%. In other words, for patients with major burns, the formal intravascular route is the preferred choice, except in mass casualty situations where access to medical care is limited, and provided the gastrointestinal tract is uninjured. In such circumstances, enteral resuscitation with balanced salt solutions can be initiated.[10,11]

Formal fluid resuscitation formulas which were introduced in the 1960s and 1970s have been used effectively all over the world.[12] The “Parkland” formula, which calculates the amount of fluid required to resuscitate a patient based on percentage-burn, remains the most commonly used formula in the United Kingdom and Ireland where 78% of all burn units use it.[13] Similarly, a recent survey of burn units in the United States and Canada revealed that 78% of units use the Parkland formula to estimate resuscitation volumes.[14]

In centres experienced with paediatric burns, formulas which are sufficient for paediatric fluid management have been developed, as the body surface area to mass ratio in children is higher than in adults and hepatic glycogen stores in young children are depleted after 12–14 hours of fasting.[15,16]

Baxter found that patients with inhalation injury required additional fluid when compared to others.[17] Pruitt reported that patients with electrical burns and those in whom resuscitation was delayed routinely also required additional fluid.[18] However, there is growing evidence that other patients with major burns also receive far more fluid than the Parkland formula recommends.[19,20] The explanation of this experience is unclear, but large volumes of resuscitation fluid are associated with increased risk of infectious complications, acute respiratory distress syndrome (ARDS), abdominal compartment syndrome and death. Pruitt has coined the term “fluid creep” to describe this phenomenon.[21]

### Formulas used for fluid management in major burns

The most commonly used formulas are the Parkland, modified Parkland, Brooke, modified Brooke, Evans and Monafo’s formulas. These formulas take into account the body weight and the burn surface area.[22] Several formulas which were specifically developed for children by paediatric burn centres have achieved equal popularity. Given below are the formulas that have been defined and modified while in use:[17,23–34]

### Parkland formula

- *Initial 24 hours*: Ringer’s lactated (RL) solution 4 ml/kg/% burn for adults and 3 ml/kg/% burn for children. RL solution is added for maintenance for children:
  - 4 ml/kg/hour for children weighing 0–10 kg
  - 40 ml/hour +2 ml/hour for children weighing 10–20 kg
  - 60 ml/hour + 1 ml/kg/hour for children weighing 20 kg or higher
  This formula recommends no colloid in the initial 24 hours.
- *Next 24 hours*: Colloids given as 20–60% of calculated plasma volume. No crystalloids. Glucose in water is added in amounts required to maintain a urinary output of 0.5–1 ml/hour in adults and 1 ml/hour in children.

### Modified Parkland formula

- *Initial 24 hours*: RL 4 ml/kg/% burn (adults)
- *Next 24 hours*: Begin colloid infusion of 5% albumin 0.3–1 ml/kg/% burn/16 per hour

### Brooke formula

- *Initial 24 hours*: RL solution 1.5 ml/kg/% burn plus colloids 0.5 ml/kg/% burn plus 2000 ml glucose in water
- *Next 24 hours*: RL 0.5 ml/kg/% burn, colloids 0.25 ml/kg/% burn and the same amount of glucose in water as in the first 24 hours

### Modified Brooke

- *Initial 24 hours*: No colloids. RL solution 2 ml/kg/% burn in adults and 3 ml/kg/% burn in children
- *Next 24 hours*: Colloids at 0.3–0.5 ml/kg/% burn and no crystalloids are given. Glucose in water is added in the amounts required to maintain good urinary output.

### Evans formula (1952)

- *First 24 hours*: Crystalloids 1 ml/kg/% burn plus colloids at 1 ml/kg/% burn plus 2000 ml glucose in water
- *Next 24 hours*: Crystalloids at 0.5 ml/kg/% burn, colloids at 0.5 ml/kg/% burn and the same amount of glucose in water as in the first 24 hours

### Monafo formula

Monafo recommends using a solution containing 250 mEq Na, 150 mEq lactate and 100 mEq Cl. The amount is adjusted according to the urine output. In the following 24 hours, the solution is titrated with 1/3 normal saline according to urinary output.

### Formulas developed for children

The formulas developed for children[35] are as follows.

### Shriner’s cincinnati

*Initial 24 hours*:

- For older children:
  Lactated Ringer’s (RL) solution 4 ml/kg/% burn +1500 ml/m^2^ total (1/2 of total volume over 8 hours, rest of the total volume during the following 16 hours)
- For younger children:
  4 ml/kg/% burn +1500 ml/m^2^ total, in the first 8 hours
  RL solution + 50 mEq NaHCO_3_
  RL solution in the second 8 hours
  5% albumin in LR solution in the third 8 hours

### Galveston

Initial 24 hours: RL 5000 ml/m^2^ burn + 2000 ml/m^2^ total (1/2 of total volume over 8 hours, rest of the total volume in 16 hours)

### Choice of fluid

The ideal burn resuscitation is the one that effectively restores plasma volume, with no adverse effects. Isotonic crystalloids, hypertonic solutions and colloids have been used for this purpose, but every solution has its advantages and disadvantages. None of them is ideal, and none is superior to any of the others.

### Isotonic crystalloids

Crystalloids are readily available and cheaper than some of the other alternatives. RL solution, Hartmann solution (a solution similar to RL solution) and normal saline are commonly used. There are some adverse effects of the crystalloids: high volume administration of normal saline produces hyperchloremic acidosis,[36] RL increases the neutrophil activation after resuscitation for haemorrhage or after infusion without haemorrhage.[37] D-lactate in RL solution containing a racemic mixture of the D-lactate and L-lactate isomers has been found to be responsible for increased production of ROS.[38] RL used in the majority of hospitals contains this mixture. Another adverse effect that has been demonstrated is that crystalloids have a substantial influence on coagulation. Recent studies have demonstrated that *in vivo* dilution with crystalloids (independent of the type of the crystalloid) resulted in a hypercoagulable state.[39–41]

Despite these adverse effects, the most commonly used fluid for burn resuscitation in the UK and Ireland is Hartmann’s solution (adult units 76%, paediatric units 75%).[13] Another study has revealed that RL is the most popular type of fluid in burn units located in USA and Canada.[14] In our burn centres located in two different regions of Turkey (Adana in the south, and Konya and Ankara in the more central zone), the initial electrolyte measurements and potassium levels guide on the choice of fluid type, but we prefer RL solution through the initial post burn 24 hours.[42]

### Hypertonic solutions

The importance of sodium ions in the pathophysiology of burn shock has been emphasised in some previous studies. The sodium shift into the cell results in cellular oedema and hypo-osmolar intravascular fluid volume. Rapid infusion of hypertonic sodium solutions has proven to increase the plasma osmolality and limit cellular oedema. Using solutions with a concentration of 250 mEq/l, Moyer *et al*. were able to achieve effective physiological resuscitation with a lower total volume when compared to isotonic solutions in the initial 24 hours.[28,29] But Huang *et al*. found that after 48 hours cumulative fluid loads of the patient groups who were treated with hypertonic solutions or RL were similar. They also demonstrated that hypertonic sodium solution resuscitation was associated with an increased incidence of renal failure and death.[43] Currently, hypertonic fluid resuscitation seems to be an attractive choice for its theoretically physiological function, but the need for close monitoring and the risk of hypernatraemia and renal failure are the main focus of debates.

### Colloids

Leakage and accumulation of plasma proteins outside the vascular compartment contributes substantially to oedema formation. The time at which the protein leakage stops has been found to differ by various authors. Baxter’s early work showed that capillary leak may persist for 24 hours post burn.[17] Carvajal,[44] as reported by Cocks *et al*., found that albumin extravasation stops 8 hours after injury. According to Demling, capillary leakage of protein ceases significantly about 12 hours following the burn.[45] Vlachou *et al*. recently showed that endothelial dysfunction and capillary leakage are present within 2 hours after burn injury and last for a median of 5 hours, much shorter than that previously described.[46] Colloids, as hyperosmotic solutions, are used to elevate the intravascular osmolality and to stop the extravasation of the crystalloids. Therefore, controversy focusses on the administration of protein-based colloids: whether to provide them or not, which solutions to use, and when to begin. Some studies have shown that colloids provide little clinical benefit when given in the first 24 hours post burn and may have some detrimental effects on pulmonary function.[47,48] The colloid versus crystalloid debate in the literature has reflected a balance of opinion; many burn clinicians avoid the use of colloids in the early post burn period. However, Cohrane *et al*. have recently demonstrated decreased mortality in patients who received albumin. Additionally, some burn clinicians reported successful resuscitation including albumin in the early post burn period with decreased volume requirements and low weight gain compared with pure crystalloid resuscitation.[49,50] O’Mara *et al*. demonstrated decreased fluid requirements and lower intraabdominal pressures with use of fresh frozen plasma in the first 48 hours following large burns (>50%).[51] Most recently, Lawrence *et al*. have found that the addition of colloid to Parkland formula rapidly reduced hourly fluid requirements, restored normal resuscitation ratios, and ameliorated fluid creep.[52]

In our burn centres located in two different regions of Turkey (Adana in the south, and Konya and Ankara in the more central zone), we avoid using human albumin solution unless blood albumin levels are under 2 g/dl. If necessary, albumin administration is started at least 5 hours after the injury. The preferred dose of albumin after the first 24 hours is 0.5–1 g/kg/% burn. In the following days, the albumin support is continued until the blood level of albumin is 3 g/dl. But decisions for each individual patient are made according to current data from monitoring parameters such as existence of oedema, urine output, central venous pressure, pulse rate, pulse oximetry, and so on.[42,53]

### Considerations for effective resuscitation

#### Antioxidant therapy

The membrane lipid peroxidation and ROS are the main components of burn shock. In addition, it is well known that the changed permeability of leukocyte membranes due to thermal injury causes an increase in serum enzyme levels. As such, it has been assumed that membrane-stabilising agents such as zinc, selenium and vitamin E could help in the recovery of burned patients.[55] Antioxidant therapy has been the interest of various studies.[54,55] In an *in vitro* study, we found that the addition of the membrane-stabilising vitamin E, zinc and selenium prevented the increase of acid phosphatase (a marker of lysosomal enzyme activity) significantly (*P* < 0.01).[55] In a prospective clinical trial in which antioxidant ascorbic acid was administered to major burn patients, the ascorbic acid group required 45% less fluid when compared to the control group.[56] Recently, Biesalski and Mc Gregor reviewed the ascorbic acid treatment in critical care patients, including those with major burns. They concluded that a significant body of pharmacological evidence and sound preliminary clinical evidence supports the biological feasibility of using the exemplary antioxidant, vitamin C, in the treatment of critically ill patients.[57]

#### Opioids and fluid resuscitation

Opioids have been the mainstay of pain control in burn patients. These drugs have a significant effect on the cardiovascular system. Use of these drugs is associated with decreased blood pressure. In a recent study, Sullivan *et al*. compared burn patient groups treated in 1975–1979 with similar patients treated in 2000.[20] This comparison emphasised that the opioid dosage correlated with the fluid requirements in these patients and fluid creep was a consequence of the increasing use of narcotics during initial burn care.

#### Monitoring

All resuscitation formulas are meant to serve as guides only. Consequently, fluid management in major burns should be monitored using clinical and laboratory parameters. In severe burns, if peripheral intravenous access cannot be achieved, central venous catheterisation or surgical vascular access must be considered. After the venous line is in place, a urinary catheter and a nasogastric tube should be inserted to control and monitor the patient’s fluid balance.[58] Hypotension is a late finding in burn shock; so, pulse rate is a much more sensitive monitoring parameter than arterial blood pressure. Fluid shifts are rapid during the early period of burn shock (24–72 hours); so, serial determinations of haematocrit, serum electrolytes, osmolality, calcium, glucose, and albumin are essential to help determine the appropriate method of fluid replacement. The best single indicator is the urine output on an hourly basis. In addition, major burn patients must be fully monitored with continuous electrocardiography, continuous respiratory rate and pulse oximetry, central venous pressure line, arterial line, foley catheter, and temperature probes. In unstable, severely burned patients and ventilated patients, capnometry, pulmonary arterial catheter or oesophageal Doppler and Doppler monitor for compartment syndromes are recommended.[59] Recently, Lawrence *et al*. suggested that measuring the hourly ratio of fluid infusion (ml/kg/% TBSA/kg) and urine output (ml/kg/hour) was an effective means of expressing and tracking fluid requirements.[52]

## BURN CARE PROCEDURES AT BAŞKENT UNIVERSITY HOSPITALS

The treatment of all patients begins at the time of hospitalisation. Following a routine examination, IV fluid (saline or saline with dextrose) is administered, and following the results of the electrolyte measurements, provided potassium levels are normal, the solution is changed to Ringer’s lactate. The rate of administration is adjusted according to urine output of at least 50 ml/hour. If the patient is oliguric and acidotic, sodium bicarbonate, 20–40 g of mannitol and 40–100 mg furosemide are given. If the patient still remains oliguric and potassium, blood urea nitrogen, and creatinine levels are rising, peritoneal or haemodialysis using a double lumen subclavian catheter is resorted. We think that this system is very easy to use for both haemodialysis and parenteral nutrition. A urinary catheter and a central venous pressure line are used only in severe cases or if clinical evaluation so indicated.

Following initial stabilisation, the patients are taken to the dressing room for re-evaluation, and if necessary, debridement, escharotomy, and fasciotomy are preformed.[60,61] Escharotomy and fasciotomy are needed when compartment syndrome is about to occur in a space that has reached up to its maximum distensibility (30 mmHg). In the case of severe flame burns and high-voltage electrical burns with suspected compartment syndrome, the incision should include the eschar and the deep fascia of each of the affected muscle compartments.[59] We perform the escharotomies or fasciotomies as an emergency. While performing escharotomies or fasciotomies, a careful haemostasis is essential in order to prevent excessive blood loss which may cause a negative effect on the fluid management of the burn shock. Wounds are cleansed and closed using one of the local chemotherapeutic agents such as silver sulphadiazine, mafenide acetate, or silver-incorporated amniotic membrane. This procedure is repeated until all nonviable tissue was removed in cases where amputation is required. Wounds are then closed with a skin graft or a flap. Rehabilitation such as physical therapy is started while patients are hospitalised and continued after discharge, if necessary.[60]

### Fluid management in electrical burns

Pruitt reported that patients with electrical burns required additional fluid.[18] In our previous study in which an 11-year experience was reported, we have found two major complications of electrical injuries: musculoskeletal involvement in 44% of patients, which required major amputation in 79%, and acute renal failure (ARF) in 14.51% of patients. In spite of treatment with peritoneal dialysis or haemodialysis, the mortality rate for patients with renal failure was quite high (59%).[61] In the light of these data, it is clear that the main threat in the initial period is the development of acute tubular necrosis and ARF related to the precipitation of myoglobin and other cellular products. Myoglobinuria is a common finding in patients with electrical injuries. The phenomenon is manifested as high-concentrated and pigmented urine. The goal is to maintain a urine output of 1–2 ml/kg/ hour until the urine clears. In non-responding patients, alkalisation of the urine and the use of osmotic agents may prevent death.[59]

### Acute renal failure and dialytic support in severe Burns

ARF is a severe complication of burns, which occurs in 0.5–30% of burn patients.[62] ARF has been found to be related to the size and depth of burns. Microalbuminuria and urinary malondialdehyde are useful markers for prediction of renal outcome in such group of patients.[63] Burn size and septicaemia proved to be the only clinical parameters that predict renal outcome.[62,63] Two forms of acute renal failure have been described in burn patients: The first form occurs in the initial few hours after injury. This form is related to hypovolemia with low cardiac output, and systemic vasoconstriction during the resuscitation period. However, this form of ARF became less frequent due to the aggressive fluid resuscitation policy at the acute stage of the burn management. The other form occurs in the second week and is related to sepsis and multiorgan failure.[62] Fluid shift, stress-related hormones, myocardial depression, inflammatory mediators and nephrotoxic agents are also supposed to be the triggers of the ARF that occurs in the second week.[64,65] Dialytic support has to be initiated in such cases. In burn patients with ARF, dialysis is indicated for fluid overload, hypercalcaemia, pulmonary oedema, unresponsiveness to diuretics, acidosis and uraemic complications. Although peritoneal dialysis is a good method, it has some complications such as low rates of ultrafiltration, respiratory problems, increased intraabdominal pressure, protein losses and bacterial or fungal peritonitis. In addition, peritoneal dialysis is contraindicated in patients with abdominal wall burns. Another choice for dialysis is conventional intermittent haemodialysis (CIHD). Although high and stable efficiency and a high rate of haemofiltration are provided by CIHD, post dialytic rebound, difficulty in balancing the solutes and cardiac arrhythmia are the most common complications. Additionally, CIHD is not suitable for severe burn patients who are hypotensive. In our burn units, we prefer to use the continuous veno-venos haemofiltration (CVVH) for the burn patients complicated with ARF. In their recent preliminary study, Sun *et al*. have also advocated that CVVH is an appropriate tool for treating ARF, with a lower incidence of vascular complications than continuous arteriovenous haemodialysis.[66]

## AMERICAN BURN ASSOCIATION PRACTICE GUIDELINES FOR BURN SHOCK RESUSCITATION

Pham *et al*. reviewed recent data in the literature to support an appropriate fluid management in burn patients, but they found that there are insufficient data in the literature for this purpose. So, they recommended a rational approach for the initial treatment of burn patients in the light of their investigations. The following are the practice guidelines for burn shock resuscitation, recommended by the American Burn Association.[11]

### Guidelines

- Adults and children with burns greater than 20% TBSA should undergo formal fluid resuscitation using estimates based on body size and surface area burned.
- Common formulas used to initiate resuscitation estimate a crystalloid need for 2–4 ml/kg body weight/% TBSA during the first 24 hours.
- Fluid resuscitation, regardless of solution type or estimated need, should be titrated to maintain a urine output of approximately 0.5–1.0 ml/kg/hour in adults and 1.0–1.5 ml/kg/hour in children.
- Maintenance fluids should be administered to children in addition to their calculated fluid requirements caused by injury.
- Increased volume requirements can be anticipated in patients with full-thickness injuries, inhalation injury and a delay in resuscitation.

### Options

- The addition of colloid-containing fluid following burn injury, especially after the first 12–24 hours postburn, may decrease the overall fluid requirements.
- Oral resuscitation should be considered in awake and alert patients with moderately sized burns and is worthy of further study.
- Hypertonic saline should be reserved for providers experienced in this approach. Plasma sodium concentrations should be closely monitored to avoid excessive hypernatraemia.
- Administration of high-dose ascorbic acid may decrease the overall fluid requirements, and is worthy of further study.

In the above-mentioned study, Pham *et al*. emphasised that the guidelines they had designed could aid especially the physicians who were responsible for the triage and initial treatment of burn patients.[11]

## CONCLUSION

Several studies have supported that patients who receive larger volumes of resuscitation fluid are at higher risk for injury complications and death. In the light of this prediction, the chosen types and rates of the fluid administration in major burns are at the focus of controversy. It must be kept on minds that these debates look for a rational approach for an adequate fluid resuscitation. Currently used guidelines are based on the various experiences all over the world, and the developing experiences will bring a new approach. So, clinicians must be aware of this vast experience and ongoing literature debates in order to improve the status of this patient group.
